# Exosomal circ_0000387 Is Associated With Ovarian Cancer Metastatic Regulation Through IGF2BP3‐Related Signalling and Endothelial VWF Responses

**DOI:** 10.1002/jbt.71135

**Published:** 2026-11-04

**Authors:** Yulian Xie, Peijun Shen, Hailin Chen, Qianqian Shen, Xiaoxia Tong

**Affiliations:** ^1^ Department of Gynaecology Qingpu Branch of Zhongshan Hospital Affiliated to Fudan University Shanghai China; ^2^ Department of Gynaecology Luodian Hospital Shanghai China

**Keywords:** circ_0000387, exosomes, IGF2BP3, ovarian cancer, VWF signaling

## Abstract

This study investigated the context‐dependent roles of circ_0000387 in ovarian cancer (OC) metastasis, focusing on its tumour‐cell‐intrinsic effects and exosome‐mediated tumour–endothelial communication. circRNA sequencing was performed on paired primary and metastatic ovarian cancer tissues to identify metastasis‐associated circRNAs. Gain‐ and loss‐of‐function experiments in SKOV3 cells assessed malignant phenotypes, while CHIRP and RIP assays examined the interaction between circ_0000387 and IGF2BP3. Exosomes were isolated and characterised, and SKOV3–endothelial cell co‐culture experiments evaluated exosome‐associated effects on endothelial VWF responses and tumour‐cell behaviour. An intraperitoneal dissemination mouse model was used for in vivo validation. circ_0000387 was expressed at higher levels in metastatic ovarian cancer tissues than in paired primary tumours. In SKOV3 cells, circ_0000387 interacted with IGF2BP3 and promoted cell viability, invasion, and migration, accompanied by AKT activation, AMPK inhibition, and increased YAP nuclear accumulation. In contrast, circ_0000387‐enriched exosomes increased endothelial VWF secretion and were associated with reduced SKOV3 invasion and migration in an FVIII‐modulated co‐culture context, accompanied by increased AMPK activation and reduced YAP nuclear accumulation. These effects were attenuated by inhibition of exosome release or VWF blockade. In vivo, circ_0000387 overexpression was associated with reduced intraperitoneal dissemination and ascites formation, and these effects were attenuated by Rab27a silencing or VWF neutralisation. circ_0000387 exhibits context‐dependent functions in ovarian cancer, promoting malignant phenotypes within tumour cells through IGF2BP3‐associated AKT/AMPK/YAP signalling while being associated with reduced metastatic activity through exosome‐dependent, VWF‐related tumour–endothelial responses. These findings identify circ_0000387 as a potential regulator of ovarian cancer metastasis warranting further mechanistic and clinical validation.

## Graphical abstract

In SKOV3 cells, circ_0000387 interacts with IGF2BP3 and is linked to AKT activation, AMPK inhibition, and increased YAP activity. Circ_0000387‐enriched exosomes increase endothelial VWF secretion in HUVECs, with reciprocal AKT/AMPK/YAP changes. In vivo, this exosome–VWF axis is associated with reduced peritoneal nodules, ascites, and SKOV3 invasion/migration, effects reversed by Rab27a knockdown or anti‐VWF.

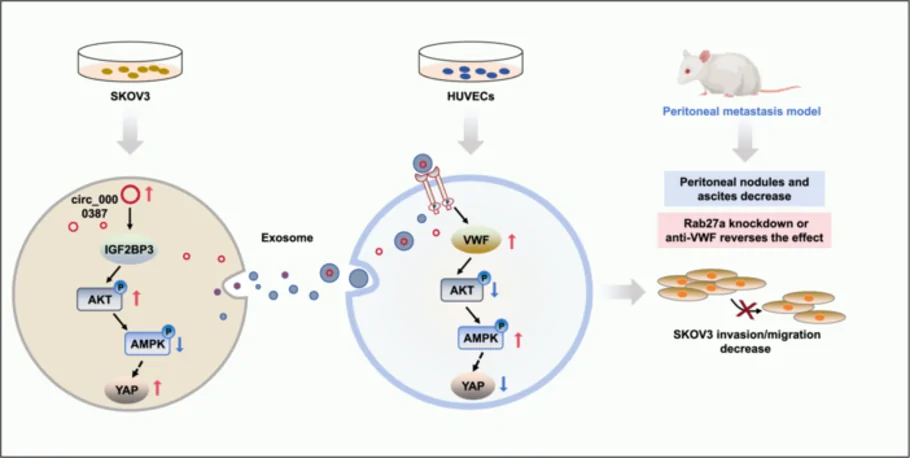

## 1 Introduction

Ovarian cancer (OC) remains the most lethal gynecologic malignancy, largely due to its aggressive metastatic behaviour and the predominance of intraperitoneal dissemination at diagnosis [1]. The metastatic spread of OC is driven by a combination of cellular and molecular processes, including enhanced tumour cell proliferation, epithelial–mesenchymal transition (EMT), extracellular matrix remodeling, and dynamic interactions between tumour cells and the peritoneal microenvironment [1]. Among these interactions, crosstalk with endothelial cells plays a pivotal role in facilitating transcoelomic implantation and metastatic colonization. In addition, several hallmark signaling pathways critically shape the metastatic phenotype of OC. The PI3K/AKT pathway enhances survival, EMT, and chemoresistance, thereby promoting peritoneal dissemination [1]. Conversely, loss of AMPK activity facilitates metabolic reprogramming and motility under stress [2]. Dysregulated YAP signaling further drives cytoskeletal remodeling and mechanotransduction, which are essential for intraperitoneal spread [3, 4]. However, the upstream non‐coding regulators coordinating these pathways remain insufficiently defined.

Circular RNAs (circRNAs) have emerged as important regulators of cancer biology due to their stability, conservation, and ability to modulate transcription, RNA–protein interactions, and signaling pathways [5, 6]. Increasing evidence indicates that circRNAs participate in tumour progression, yet the specific circRNAs that govern OC metastasis and their molecular mechanisms remain largely unexplored. In particular, how circRNAs integrate into key oncogenic pathways—such as AKT, AMPK, and YAP signaling, which regulate cellular metabolism, proliferation, migration, and mechanotransduction—remains poorly understood [7, 8]. Among upstream modulators of these pathways, the RNA‐binding protein IGF2BP3 stabilizes multiple pro‐metastatic transcripts and is frequently upregulated in aggressive OC [9, 10]. Yet, whether circRNAs regulate IGF2BP3‐dependent signaling in OC is largely unknown.

Another critical but underappreciated layer of metastasis regulation involves extracellular vesicles, especially exosomes. Tumour‐derived exosomes can carry bioactive cargos, including circRNAs, and mediate intercellular communication between cancer cells and stromal components of the tumour microenvironment [11, 12]. Tumour–endothelial communication is increasingly recognised as an important determinant of metastatic dissemination. Among endothelial‐derived factors, von Willebrand factor (VWF), a multimeric glycoprotein predominantly released by endothelial cells, has functions beyond haemostasis and has been implicated in endothelial activation, vascular permeability, tumour‐cell adhesion, transendothelial migration, and metastatic progression [13, 14]. Tumour‐derived signals can stimulate endothelial VWF release, and emerging evidence further indicates that small extracellular vesicle‐associated VWF participates in reciprocal tumour–endothelial communication and metastatic regulation [15]. VWF also serves as the principal carrier and stabilising protein for coagulation factor VIII (FVIII), while FVIII has increasingly been implicated in cancer‐associated pathophysiology [13, 16]. These observations provide a biological rationale for examining VWF as an endothelial response marker and for further exploring whether FVIII modifies VWF‐associated responses to tumour‐derived exosomes. However, whether exosomal circRNAs regulate these endothelial responses in OC remains unclear.

Despite increasing evidence for the roles of circRNAs in cancer progression, the circRNA‐mediated mechanisms linking tumour‐intrinsic signalling to tumour–endothelial communication in OC remain incompletely understood. AKT, AMPK, and YAP signalling are key components of oncogenic signalling networks and exhibit extensive crosstalk in cancer progression [17]. IGF2BP3‐mediated RNA regulation has also been implicated in ovarian cancer progression [9, 10]. Separately, endothelial VWF responses have been linked to tumour–endothelial interactions and metastatic regulation [13, 14, 15]. Whether circRNAs coordinately influence these processes in OC, however, remains unclear. Accordingly, we performed circRNA profiling to identify candidate circRNAs associated with OC.

Therefore, this study aimed to (i) determine the cell‐intrinsic effects of the selected candidate circ_0000387 on OC malignant phenotypes and associated signalling pathways, and (ii) investigate whether exosomal circ_0000387 modulates endothelial VWF responses and whether FVIII influences the associated tumour–endothelial effects. By delineating these mechanisms, we sought to clarify the context‐dependent roles of circ_0000387 in ovarian cancer progression and tumour–endothelial communication.

## 2 Material and Methods

### 2.1 Clinical Sample Collection

A total of 20 patients with ovarian cancer (OC) were enrolled at the Qingpu Branch of Zhongshan Hospital Affiliated to Fudan University. Paired primary ovarian tumour tissues and corresponding metastatic tissues were obtained from the same patients, yielding 20 matched pairs of tissue specimens. Written informed consent was obtained from all participants. The study was approved by the Ethics Committee of the Qingpu Branch of Zhongshan Hospital Affiliated to Fudan University (Approval No. Qingyi2022‐39) and was conducted in accordance with the Declaration of Helsinki.

Patients were eligible if they had histologically confirmed epithelial ovarian cancer and available paired primary and metastatic tumour tissues for analysis. Patients with malignancies originating outside the ovary or with additional primary tumours were excluded. Clinical information, including age, FIGO stage, and relevant comorbidities, was collected. The baseline clinical characteristics of the study population are summarised in Table 1.

**Table 1:** Baseline clinical characteristics of patients with ovarian cancer.

Characteristic	Patients with ovarian cancer (*n* = 20)
Pretreatment FIGO stage	IIIb, *n* = 9; IIIc, *n* = 11
Age, years	42–62 (mean 53)
Hypertension, *n*	13
Diabetes mellitus, *n*	11
Hyperlipidaemia, *n*	15

### 2.2 CircRNA Sequencing

RNA sequencing and bioinformatics analysis were performed by Major Bio Co. Ltd. (Shanghai, China). CircRNA sequencing was performed using four matched pairs of primary and metastatic ovarian cancer tissues selected from the clinical cohort. Total RNA was extracted from metastatic and primary ovarian cancer tissues using TRIzol reagent. RNA quality and integrity were assessed using a NanoDrop spectrophotometer and Agilent 2100 Bioanalyzer. Ribosomal RNA was removed prior to library construction, and sequencing was performed on an Illumina platform with paired‐end reads.

Raw reads were filtered to remove low‐quality sequences and aligned to the human reference genome (GRCh38). CircRNAs were identified using CIRCexplorer2 based on back‐splice junction reads. Differential expression analysis was performed using DESeq. 2, and circRNAs with fold change (FC) > 2 and *p* < 0.05 were considered significantly dysregulated. Heatmaps and volcano plots were used to show the different circRNAs. KEGG analyses were used to identify related signaling pathways.

### 2.3 Fluorescence in Situ Hybridization (FISH)

FISH was performed to determine the subcellular localization of circ_0000387 in SKOV3 cells according to the manufacturer's protocol (Service Bio Co. Ltd., Wuhan, China). Images were acquired from five randomly selected fields using a confocal laser microscope (Leica, Germany) and analyzed with ImageJ software.

### 2.4 Cell Culture and Transfection

Human ovarian cancer cell lines SK‐OV‐3 (female, ovarian adenocarcinoma; RRID: CVCL_0532), OVCAR3 (female, ovarian adenocarcinoma; RRID: CVCL_0465), CaOV3 (female, ovarian adenocarcinoma; RRID: CVCL_0201), A2780 (female, ovarian endometrioid adenocarcinoma; RRID: CVCL_0134), and human umbilical vein endothelial cells (HUVECs; human; umbilical vein endothelium; RRID: CVCL_2959) were obtained from the Cell Bank of the Chinese Academy of Sciences (Shanghai, China). Cell identity was verified based on the short tandem repeat (STR) profiling certificates provided by the supplier before use. All cell lines were routinely tested and confirmed to be free of mycoplasma contamination before and during the experiments. For baseline expression analysis, circ_0000387 levels were examined in SKOV3, OVCAR3, CaOV3, and A2780 cells by qRT‐PCR. Conditioned media were collected from the corresponding cell cultures, and exosomes were isolated as described below. Exosomal RNA was subsequently extracted, and exosomal circ_0000387 levels were quantified by qRT‐PCR. Cells were cultured under the recommended conditions in medium supplemented with 10% fetal bovine serum at 37°C in a humidified incubator containing 5% CO_2_. Exosome‐depleted FBS was used where indicated. SKOV3 cells were used for subsequent functional and mechanistic experiments. According to the experimental design, SKOV3 cells were transfected with OE‐NC, OE‐circ_0000387, si‐NC, si‐circ_0000387, si‐Rab27a, or OE‐IGF2BP3 using lentiviral vectors or siRNAs provided by Sangon Biotech, while HUVECs were transfected with si‐NC or si‐VWF for endothelial functional assays. Transfection was performed at 70–80% confluence, and rescue interventions were applied as indicated, including Compound C (10 μM) or the exosome‐release inhibitor GW4869 (25 μM). For co‐culture experiments, transfected SKOV3 cells and HUVECs were cultured in Transwell systems to evaluate exosome‐associated effects on endothelial VWF secretion and SKOV3 phenotypes under the indicated genetic or pharmacological conditions.

### 2.5 Animal Modeling

NOD/SCID female mice (6–8 weeks old) were used to establish an intraperitoneal ovarian cancer dissemination model. This model was selected because intraperitoneal injection of SKOV3 cells reliably induces peritoneal implantation, ascites formation, and metastatic nodule development, closely mimicking the clinical pattern of human ovarian cancer, as demonstrated in previous studies employing similar xenograft strategies [18]. In brief, mice received an intraperitoneal injection of 1 × 10^6^ SKOV3 cells suspended in PBS. A total of 20 mice were randomly assigned into four groups (*n* = 5 per group): Control (SKOV3 only), OE‐circ (SKOV3 with circ_0000387 overexpression), OE‐circ+si‐Rab27a, and OE‐circ+anti‐VWF. Randomization was performed using a computer‐generated random number sequence, and all animal procedures were conducted blinded to group allocation. The investigator responsible for tumour burden assessment and sample analysis was blinded to the treatment groups. To evaluate the contribution of exosome secretion and endothelial VWF signaling, a subset of mice additionally received intraperitoneal administration of an anti‐VWF neutralizing antibody (5 μg every 3 days), following an established dosing regimen shown to effectively suppress VWF activity in vivo [15]. Animals were maintained for 4 weeks to allow peritoneal dissemination before termination and subsequent assessment of tumour burden. Tumour burden was quantitatively assessed by (i) counting all visible peritoneal metastatic nodules under a dissecting microscope, and (ii) measuring ascites volume using a graduated syringe. For histological and molecular analysis, representative tumour nodules from the peritoneum and omentum were excised, fixed in 10% neutral‐buffered formalin, embedded in paraffin, and sectioned at 4–5 μm. Sections were subjected to immunohistochemical staining for YAP, followed by hematoxylin counterstaining. Staining intensity was evaluated under a light microscope, and representative images were captured for analysis.

### 2.6 CCK8 Assay

SKOV3 cells transfected with the indicated constructs were seeded into 96‐well plates and cultured for 24, 48, or 72 h. At each time point, the culture medium was replaced with serum‐free medium containing 10 μL CCK‐8 solution (No. C6005, NCM Biotech, China), followed by incubation for 3 h at 37°C. Optical density (OD) was measured at 450 nm using a microplate reader. Cells transfected with the corresponding negative controls were used as controls.

### 2.7 Co‐Culture Assay

HUVECs (1 × 10^6^ cells/well) were seeded in the lower chambers of 6‐well Transwell plates containing 2 mL of culture medium, while SKOV3 cells (5 × 10^5^ cells/well) were seeded in the upper inserts (0.4 μm pore size; Corning, USA) in 800 μL of culture medium. The cells were co‐cultured for 24 or 48 h under the indicated experimental conditions. At the specified time points, conditioned medium, HUVECs, and SKOV3 cells were separately collected for subsequent analyses.

### 2.8 Exosome Isolation

Extracellular vesicle‐enriched fractions were isolated from conditioned media of SKOV3, OVCAR3, CaOV3, and A2780 cells by differential ultracentrifugation. Cells were cultured for 24–48 h in medium containing 10% exosome‐depleted FBS before collection of conditioned media. Conditioned media were then filtered through a 0.22 μm membrane and ultracentrifuged at 100,000 × g for 70 min at 4°C. Pellets were washed once with PBS and subjected to a second ultracentrifugation at 100,000 × g for 70 min. The final pellets were resuspended in 50–100 μL PBS.

For baseline circ_0000387 expression analysis, vesicle‐enriched preparations were obtained from conditioned media of SKOV3, OVCAR3, CaOV3, and A2780 cells under comparable culture and collection conditions. Total RNA was extracted from each preparation, and equal amounts of RNA were used for reverse transcription and qRT‐PCR analysis of circ_0000387. SKOV3‐derived exosome preparations used in subsequent functional experiments were further characterised by transmission electron microscopy, nanoparticle tracking analysis, and western blotting for TSG101 and CD63. Exosome yield for the functional experiments was quantified by nanoparticle tracking analysis (particle number per mL) and micro‐BCA protein assay. Equal exosome inputs were normalized to 1 × 10^9^ particles, with corresponding protein amounts within the range of 0.8–1.2 μg per condition. TEM and NTA analyses of SKOV3‐derived exosomes were performed using three independent preparations (*n* = 3 biological replicates).

### 2.9 Exosome Labelling and Uptake Assay

SKOV3‐derived exosomes were fluorescently labelled with PKH67 (C3635S, Beyotime, China) according to the manufacturer's instructions. Excess unbound dye was removed before labelled exosomes were incubated with HUVECs. Cells were then washed, fixed, counterstained with DAPI, and examined by confocal fluorescence microscopy to visualise exosome‐associated fluorescence signals.

### 2.10 ExoELISA

Relative CD63‐associated exosome abundance in culture supernatants was assessed using an ExoELISA Complete Kit for CD63 detection (Cat. No. EXOEL‐CD63A‐1, System Biosciences, USA) according to the manufacturer's instructions. Equal volumes of conditioned medium collected under comparable culture conditions and over the same incubation period were used as inputs. Absorbance was measured using a microplate reader, and ExoELISA signals were expressed as relative CD63‐associated exosome abundance in conditioned medium.

### 2.11 ELISA

VWF antigen levels in HUVEC culture supernatants and mouse plasma were measured using a commercially available ELISA kit (DY2764‐05, R&D Systems, USA) according to the manufacturer's instructions. Culture supernatants were collected after the indicated treatments and clarified by centrifugation before analysis. Mouse plasma samples were collected at the experimental endpoint and analysed using the same assay. Absorbance was measured using a microplate reader, and VWF concentrations were calculated from the corresponding standard curve.

### 2.12 RNase *R* Treatment

Total RNA extracted from SKOV3 cells was incubated with RNase R (R7092S, Beyotime, China) according to the manufacturer's instructions. Equal amounts of untreated RNA served as controls. Residual circ_0000387 and its linear host transcript CCDC91 were subsequently quantified by qRT‐PCR.

### 2.13 qRT‐PCR

Total RNA was isolated from cells and tissues using TRIzol reagent (Servicebio Wuhan, China) according to the manufacturer's protocol. Exosomal circRNA was extracted using the commercial reagent kit (ABSIN, China, Shanghai absin Biotechnology Co). Total RNA was reverse transcribed into cDNA using the PrimeScript Reverse Transcriptase Kit (Takara, Dalian, China) according to the manufacturer's protocol. SYBR Green Master Mix (Takara, Dalian, China) was used for qRT‐PCR analysis on the CFX96 real‐time fluorescence polymerase chain reaction system (Bio‐Rad). The primer sequence was designed and provided by Shanghai Sangon Biotech Co. Ltd. (Shanghai, China). The primer sequence is shown in Table 2. The 2^−ΔΔCt^ method was used to quantify the relative expression of the target genes.

**Table 2:** The primer sequences.

Primers	Forward	Reverse
GAPDH	5'‐GGAAGCTTGTCATCAATGGAAATC ‐3'	5'‐ TGATGACCCTTTTGGCTCCC ‐3'
circ_0000387	5'‐AAATGCATAGACAGTTAGAAATTCTAATT‐3'	5'‐TACCATTACTCACAATTTTAGCAATCT‐3'
CCDC91	5'‐GCTAGATACAGAGAAGGAACTG‐3'	5'‐AGGAGAGGAACAGTTCCAAAC‐3'

### 2.14 Western Blot Analysis

Cells or tissue samples were lysed on ice using lysis buffer (Beyotime, Shanghai, China), and protein concentrations were determined using a BCA protein assay kit (Beyotime, Shanghai, China). Equal amounts of protein were separated by 10% SDS‐PAGE and transferred to PVDF membranes (PG112‐10, Shanghai Yichen Biotechnology Co, China). After blocking, the membrane was incubated with the appropriate primary antibodies at 4°C overnight, including anti‐AMPK (ab32047, 1:5000, Abcam, UK), anti‐p‐AMPK (ab133448, 1:10000, Abcam, UK), anti‐AKT (#9272, 1:1000, CST, USA), anti‐p‐AKT (ab38449, 1:1000, Abcam, UK), anti‐IGF2BP3 (ab177477, 1:1000, Abcam, UK), anti‐YAP (1:1000, ab205270, Abcam, UK), anti‐p‐YAP (1:2000, AF3328, Affinity Biosciences, USA), anti‐Histone H3 (1:5000, ab1791, Abcam, UK), anti‐Rab27a (1:1000, ab55667, Abcam, UK), anti‐TSG101 (67381‐1‐Ig, 1:1000, Proteintech, China), anti‐CD63 (25682‐1‐AP, 1:1000, Proteintech, China), and anti‐GAPDH (internal reference, 81640‐5‐RR, 1:10000, Proteintech, China). The membrane was then incubated with HRP‐conjugated secondary antibody IgG (ab6721, 1:5000, Abcam) for 2 h at room temperature. Signals were visualized using an ECL detection kit (Bio‐Rad, USA), and band intensities were quantified using ImageJ software (V1.48, National Institutes of Health, USA).

### 2.15 Nuclear and Cytoplasmic Protein Extraction

For analysis of YAP subcellular distribution, nuclear and cytoplasmic proteins were extracted according to the manufacturer's instructions. Briefly, cells were fractionated to obtain nuclear and cytoplasmic extracts separately, which were then subjected to Western blot analysis. Histone H3 and GAPDH were used as nuclear and cytoplasmic loading controls, respectively.

### 2.16 Transwell Assay

SKOV3 cells overexpressing circ_0000387 and the corresponding negative‐control (oeNC) cells were seeded in the upper chambers of Matrigel‐coated 8‐μm‐pore Transwell invasion inserts (Cat. No. 354480, Corning, USA) at a density of 6 × 10^4^ cells/well, while HUVECs were seeded in the lower chambers. For experiments assessing the effects of FVIII, recombinant human FVIII (RayBiotech, Cat. No. 228‐10436, USA) was added to the co‐culture system at final concentrations of 10 or 30 μg/mL, with the no‐FVIII condition serving as the 0 μg/mL control. For experiments combining FVIII with inhibition of exosome release, FVIII was used at 30 μg/mL and GW4869 was applied as described above. After 48 h of co‐culture, non‐invaded cells remaining on the upper surface of the membrane were gently removed, whereas cells that had invaded through the Matrigel‐coated membrane to the lower surface were stained with Diff‐Quik solution (Sysmex, Kobe, Japan). Four randomly selected microscopic fields per membrane were photographed and quantified, and the mean number of invaded cells was used as the value for each experimental replicate.

### 2.17 Wound Healing

HUVECs were seeded in the upper chambers of 0.4‐μm‐pore Transwell inserts (Corning, USA), while SKOV3 cells transfected with OE‐circ_0000387 or the corresponding negative control (oeNC) were seeded in the lower chambers at a density of 6 × 104 cells/well. For the FVIII/GW4869 experiments, recombinant human FVIII was used at 30 μg/mL, and GW4869 was applied at 25 μM as indicated. When SKOV3 cells reached approximately 90% confluence, a linear scratch was generated using a sterile pipette tip, and images were captured immediately (0 h). After 24 h of co‐culture at 37°C, the same fields were photographed again under a light microscope at ×100 magnification. Cell migration was quantified based on the change in wound width or wound closure relative to 0 h.

### 2.18 CHIRP

The RNA pulldown assay was performed using an RNA antisense purification kit (RUQIBio, Guangzhou, China) according to the manufacturer's protocol. Briefly, a total of 2 × 10^7^ cells were lysed with lysis buffer and centrifuged. The supernatant was incubated with 40 pmol of biotinylated circ0000387 probe for 3 h at 37°C. Twenty microlitres of streptavidin beads were washed and hybridized to the supernatant for 30 min at room temperature. The enriched proteins bound to the beads were eluted and used for western blot detection.

### 2.19 RIP

Cells were harvested, re‐suspended in 1 mL lysis buffer containing a protease inhibitor cocktail and RNase inhibitor. After centrifuging at 13,000 rpm for 10 min at 4°C, the supernatant was incubated with 30–40 μL Protein A‐Sepharose beads (Geneseed, Guangzhou, China) and 2 μg primary antibodies for 4 h at 4°C, followed by washing with ice‐cold 1 × PBS. The beads were incubated with Proteinase K (Geneseed, Guangzhou, China) using Trizol reagent (Geneseed, Guangzhou, China), and the purified RNA was subjected to qRT‐PCR analysis.

### 2.20 Immunofluorescence

Cells were fixed with 4% paraformaldehyde, permeabilised with Triton X‐100, and blocked with an appropriate blocking solution. Samples were incubated with primary antibodies against YAP at 4°C overnight, followed by incubation with the corresponding fluorescence‐conjugated secondary antibodies. Nuclei were counterstained with DAPI. Images were acquired using a confocal laser‐scanning microscope (LSM 780 with Airyscan; Carl Zeiss, Oberkochen, Germany). YAP subcellular localisation was quantified from randomly selected fields using ImageJ.

### 2.21 Statistical Analysis

Data analysis was performed using SPSS 26.0 (IBM‐SPSS Inc., Chicago, IL, USA). Data are presented as mean ± standard deviation (SD), unless otherwise indicated. For paired comparisons between primary ovarian tumour tissues and corresponding metastatic tissues from the same patients, a paired Student's *t*‐test was used for normally distributed data; otherwise, the Wilcoxon signed‐rank test was applied. Comparisons between two independent groups were performed using an unpaired Student's *t*‐test. Comparisons among three or more independent groups were performed using one‐way analysis of variance (ANOVA) followed by Tukey's post hoc test. For experiments involving two independent factors, including treatment‐by‐time effects in CCK‐8 assays and circ_0000387 condition‐by‐FVIII concentration effects, two‐way ANOVA was performed, followed by Tukey's multiple‐comparison test where appropriate. All cell‐based experiments were performed using three independent biological replicates unless otherwise specified. All tests were two‐sided, and *p* < 0.05 was considered statistically significant.

## 3 Results

### 3.1 Transcriptomic Profiling Identifies Metastasis‐Associated Circrna Alterations in Ovarian Cancer

KEGG enrichment analysis revealed that the differentially expressed circRNAs between metastatic and paired primary ovarian cancer tissues were associated with multiple cancer‐related signalling pathways (Figure 1A–B). Enriched pathways included endocrine resistance, central carbon metabolism in cancer, focal adhesion, AMPK signalling, Hippo signalling, PI3K–Akt signalling, and other pathways related to tumour progression and cellular metabolism. Hierarchical clustering analysis showed distinct circRNA expression patterns between metastatic and paired primary ovarian cancer tissues (Figure 1C). Multiple circRNAs showed differential relative expression between the two tissue groups, with red indicating relatively higher expression and blue indicating relatively lower expression after expression scaling. The volcano plot further identified circRNAs that were significantly differentially expressed according to the predefined fold‐change and P‐value thresholds (Figure 1D), while the scatter plot illustrated the overall distribution of upregulated, downregulated, and unchanged circRNAs (Figure 1E). Based on the differential expression analysis, circ_0000387 was selected as a candidate for subsequent validation and functional investigation.

**Figure 1:**
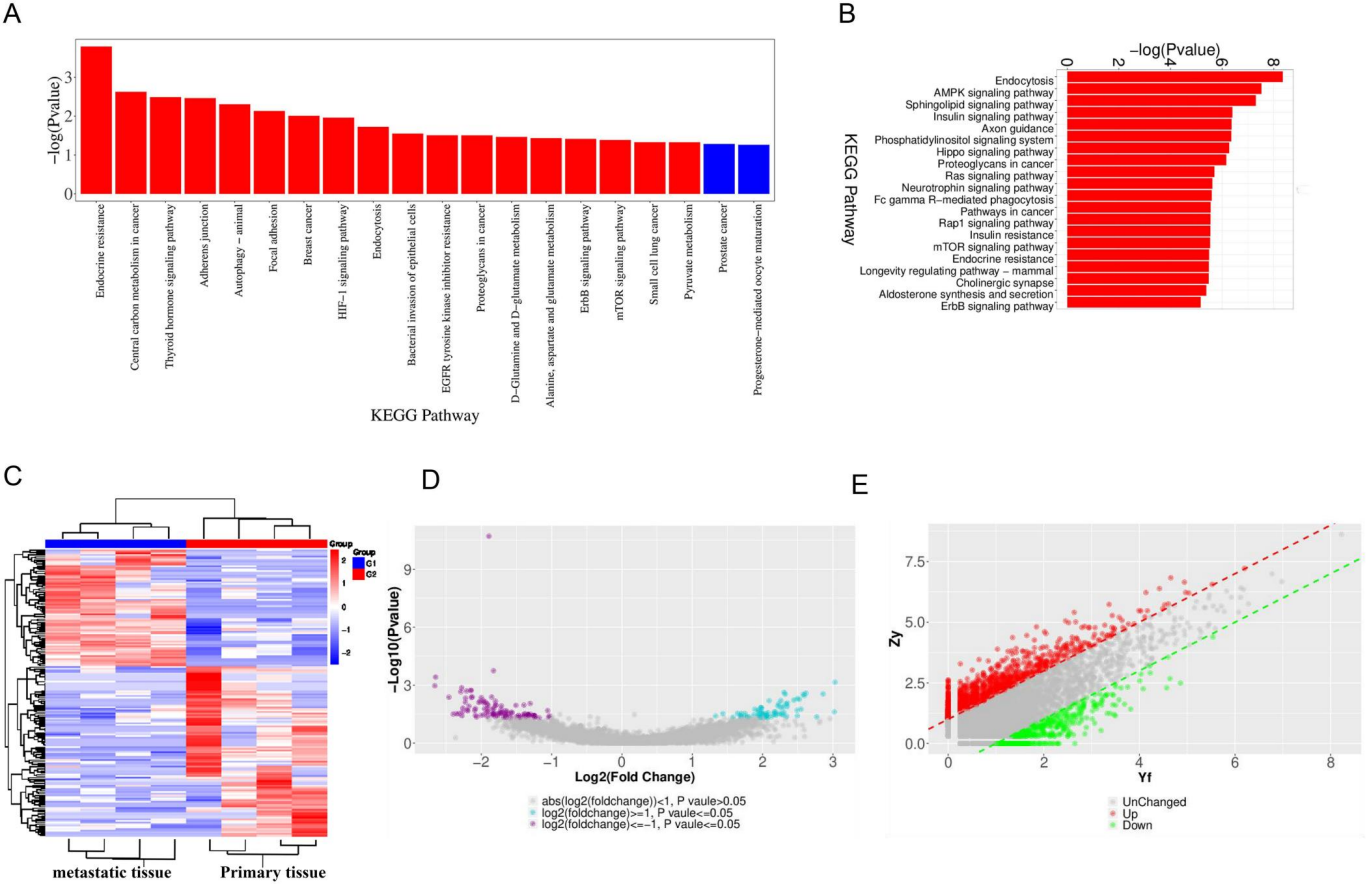
Differential circRNA expression profiling between metastatic and primary ovarian cancer tissues. (A) KEGG enrichment analysis of pathways associated with differentially expressed circRNAs. (B) Ranking of enriched KEGG pathways according to −log10(P value). (C) Hierarchical clustering heatmap of differentially expressed circRNAs. The four columns on the left represent metastatic ovarian cancer tissues, and the four on the right represent paired primary ovarian cancer tissues. The bottom annotation indicates tissue group. Red and blue indicate relatively higher and lower scaled expression, respectively. (D) Volcano plot showing differentially expressed circRNAs according to log2 fold change and statistical significance. (E) Scatter plot showing the distribution of upregulated, downregulated, and unchanged circRNAs.

### 3.2 Baseline Cellular and Exosomal circ_0000387 Expression Across Ovarian Cancer Cell Lines

Baseline circ_0000387 expression was examined in SKOV3, OVCAR3, CaOV3, and A2780 cells. qRT‐PCR showed that circ_0000387 was detectable in all four cell lines, with relatively high expression in SKOV3 and OVCAR3 cells and significantly lower levels in CaOV3 and A2780 cells (Figure S1A). A similar pattern was observed in exosomes derived from these cell lines, with higher circ_0000387 levels in SKOV3‐ and OVCAR3‐derived exosomes than in those derived from CaOV3 and A2780 cells (Figure S1B). Based on the relatively high cellular and exosomal circ_0000387 expression in SKOV3 cells, together with the suitability of SKOV3 for the subsequent functional and intraperitoneal dissemination assays, SKOV3 was retained as the principal model for mechanistic investigation.

### 3.3 circ_0000387 Modulates Cell Viability, Invasion, Migration, YAP Subcellular Distribution, and AKT/AMPK‐Related Signaling in SKOV3 Cells

RNase R digestion supported the circular nature of circ_0000387, as circ_0000387 expression remained relatively stable whereas the linear transcript CCDC91 was markedly reduced (*p* < 0.01, Figure 2A). qRT‐PCR showed that circ_0000387 levels were significantly increased in the oe‐circ group compared with the Blank and oeNC groups (*p* < 0.001), whereas si‐circ transfection significantly reduced circ_0000387 expression relative to siNC (*p* < 0.001) (Figure 2B). CCK‐8 assays further showed that cell viability was increased in the oe‐circ group but decreased in the si‐circ group compared with their respective controls (*p* < 0.001, Figure 2C).

**Figure 2:**
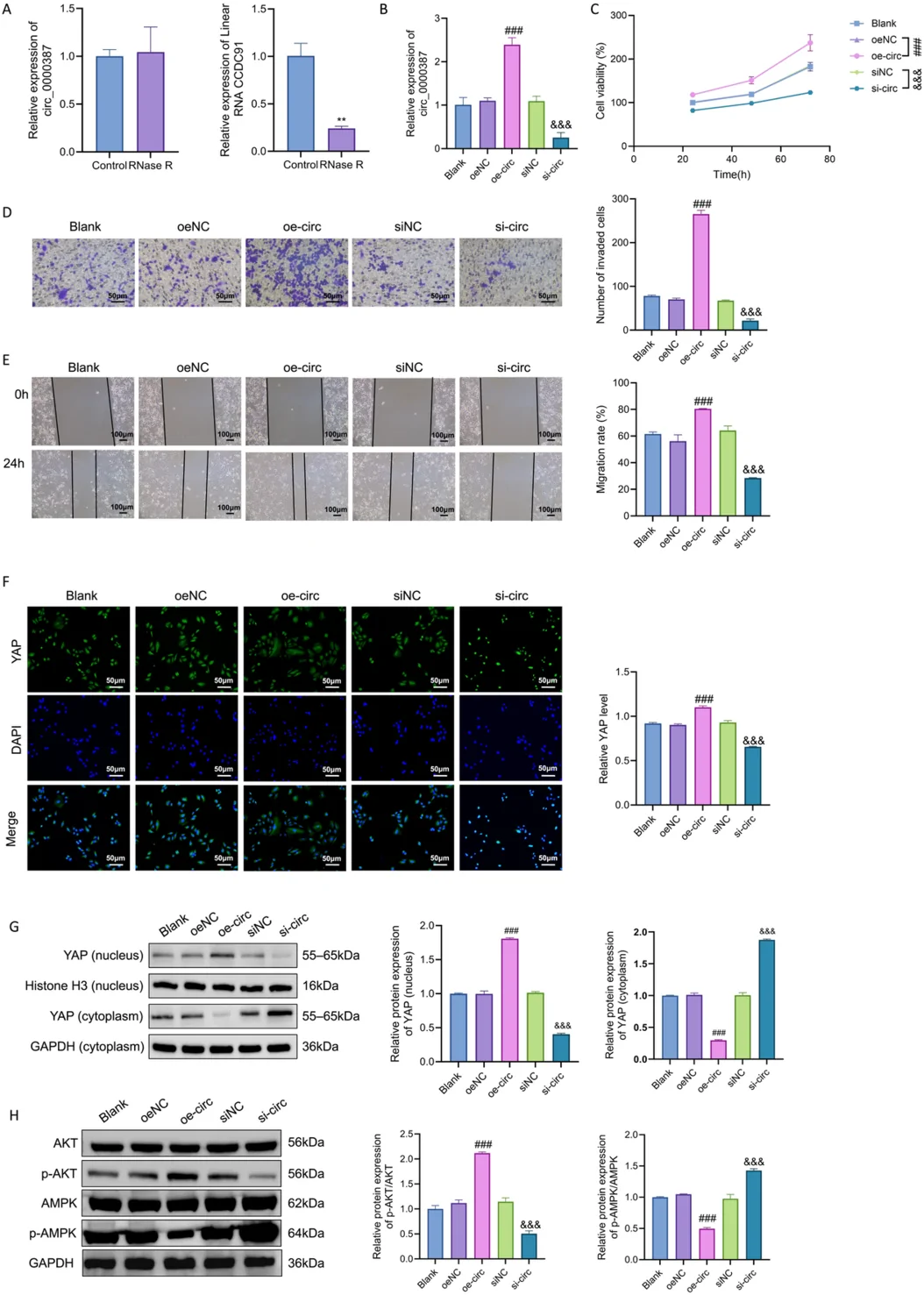
circ_0000387 modulates SKOV3 cell viability, invasion, migration, YAP nuclear distribution, and AKT/AMPK signaling. (A) qRT‐PCR analysis of circ_0000387 and linear CCDC91 following RNase R treatment. (B) circ_0000387 expression levels in Blank, oeNC, oe‐circ, siNC, and si‐circ groups. (C) CCK‐8 assay evaluating cell viability across groups. (D) Representative images and quantification of Transwell invasion. Scale bar = 50 μm. (E) Wound‐healing assay assessing migration. Scale bar = 100 μm. (F) Immunofluorescence analysis of YAP nuclear and cytoplasmic localization. Scale bar = 50 μm. (G) Western blot analysis of nuclear and cytoplasmic YAP in the indicated groups. (H) Western blot analysis of AKT, p‐AKT, AMPK, and p‐AMPK protein levels. **p < 0.01 versus Control; ^###^ *p* < 0.001 versus oeNC; ^&&&^ *p* < 0.001 versus siNC.

Transwell assays showed that circ_0000387 overexpression significantly increased invasive capacity, whereas circ_0000387 silencing reduced invasion compared with their respective controls (*p* < 0.001, Figure 2D). Consistently, wound‐healing assays demonstrated enhanced migratory capacity in the oe‐circ group and reduced migration in the si‐circ group (*p* < 0.001, Figure 2E).

Immunofluorescence analysis showed increased YAP nuclear accumulation in the oe‐circ group and reduced nuclear YAP in the si‐circ group (*p* < 0.001, Figure 2F). Subcellular fractionation followed by western blotting further supported these findings, showing increased nuclear YAP and decreased cytoplasmic YAP in the oe‐circ group, whereas the opposite pattern was observed in the si‐circ group (both *p* < 0.001, Figure 2G). These results indicate that circ_0000387 is associated with enhanced YAP nuclear accumulation in SKOV3 cells. Western blot analysis further showed that circ_0000387 overexpression was accompanied by increased p‐AKT and decreased p‐AMPK levels, whereas circ_0000387 silencing produced the opposite pattern (both *p* < 0.001, Figure 2H). Total AKT and AMPK levels remained largely unchanged across groups.

### 3.4 circ_0000387 Associates With IGF2BP3 and Modulates AKT/AMPK Signalling

FISH analysis showed that circ_0000387 predominantly exhibited cytoplasmic localization in SKOV3 cells (Figure 3A). CHIRP assays demonstrated a clear enrichment of IGF2BP3 protein in the circ_0000387 probe–captured fraction relative to bead control (Figure 3B). RIP assays further confirmed the interaction, showing significantly higher circ_0000387 RNA abundance in the IGF2BP3 immunoprecipitated fraction compared with the IgG group (*p* < 0.01, Figure 3C).

**Figure 3:**
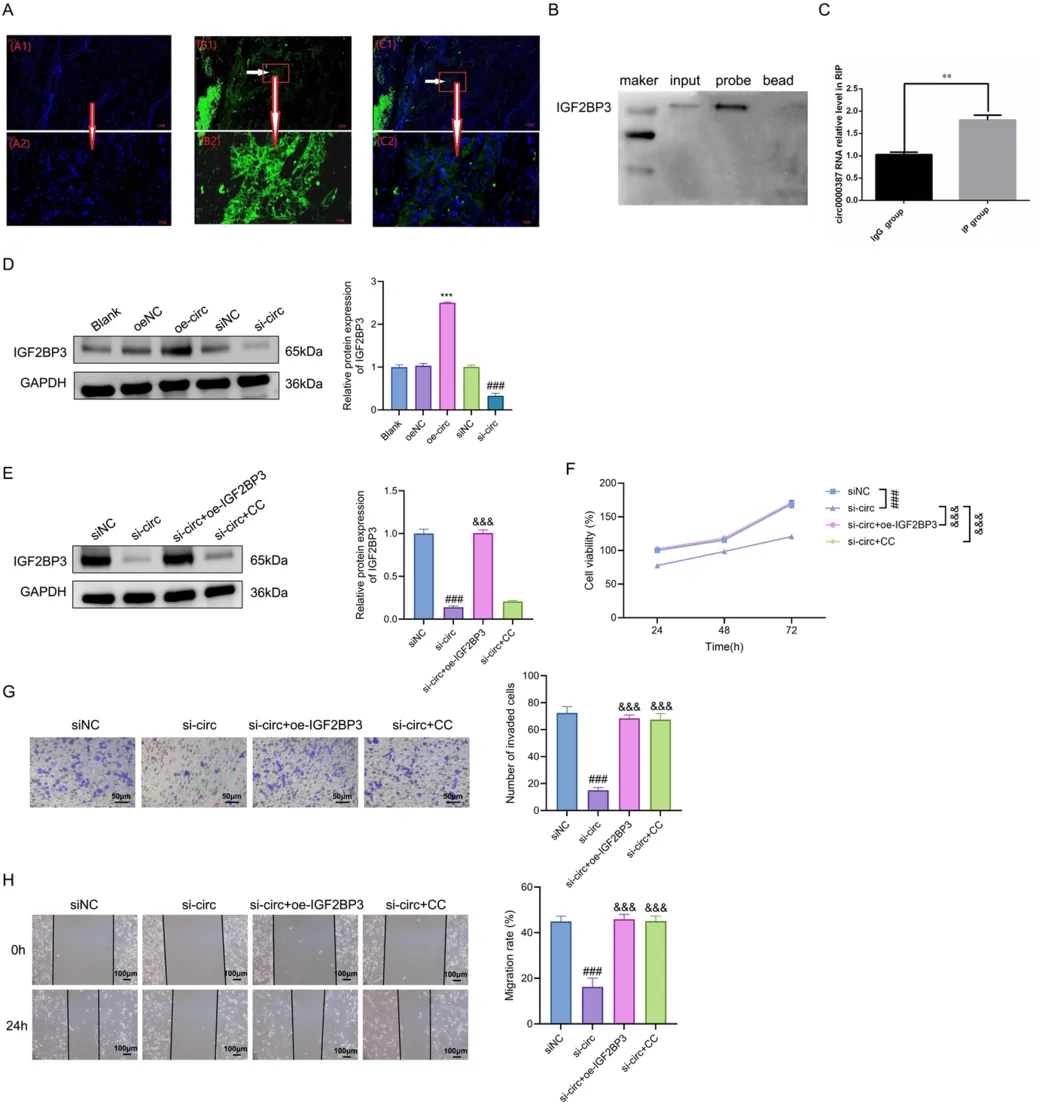
circ_0000387 interacts with IGF2BP3 and modulates AKT/AMPK signalling. (A) FISH analysis showing subcellular localization of circ_0000387. (B) CHIRP assay detecting IGF2BP3 protein enriched by circ_0000387 probe pull‐down. (C) RIP assay quantifying circ_0000387 RNA in IGF2BP3 versus IgG immunoprecipitates. (D) Western blot analysis of IGF2BP3 protein in Blank, oeNC, oe‐circ, siNC, and si‐circ groups. (E) IGF2BP3 expression in siNC, si‐circ, si‐circ+oe‐IGF2BP3, and si‐circ+Compound C groups. (F) CCK‐8 assay evaluating cell viability in corresponding groups. (G) Transwell invasion assay. Scale bar = 50 μm. (H) Wound‐healing migration assay. Scale bar = 100 μm. **p < 0.01, ***p < 0.001 versus Control; ^###^ *p* < 0.001 versus siNC; ^&&&^ *p* < 0.001 versus si‐circ.

In SKOV3 cells, western blotting showed elevated IGF2BP3 protein levels in the oe‐circ group compared with Blank and oeNC groups, whereas si‐circ led to a marked decrease relative to siNC (Figure 3D, *P* < 0.001). In rescue experiments, IGF2BP3 expression was reduced in si‐circ cells but restored by IGF2BP3 overexpression (*p* < 0.001), while Compound C treatment resulted in only a partial increase (Figure 3E).

CCK‐8 assays revealed that si‐circ significantly decreased cell viability compared with siNC. Both IGF2BP3 overexpression and Compound C treatment effectively rescued this reduction, restoring cell viability to levels comparable to siNC (Figure 3F, *p* < 0.001). Similarly, Transwell assays showed markedly reduced invasion in si‐circ cells, whereas both IGF2BP3 overexpression and Compound C treatment effectively rescued this reduction (Figure 3G, *p* < 0.001). Wound‐healing assays demonstrated reduced migration in si‐circ cells compared with siNC, and both IGF2BP3 overexpression and Compound C enhanced migration relative to si‐circ (Figure 3H, *P* < 0.001).

### 3.5 circ_0000387 Is Enriched in Exosomes and Its Secretion Is Regulated by Rab27a

Transmission electron microscopy showed round, membrane‐bound vesicles consistent with exosomes (Figure 4A, left). Nanoparticle tracking analysis demonstrated a predominant peak around 100 nm, confirming the expected size distribution of isolated vesicles (Figure 4A, right). Western blot analysis detected the exosomal markers TSG101 and CD63 in vesicles derived from SKOV3 culture supernatants (Figure 4B).

**Figure 4:**
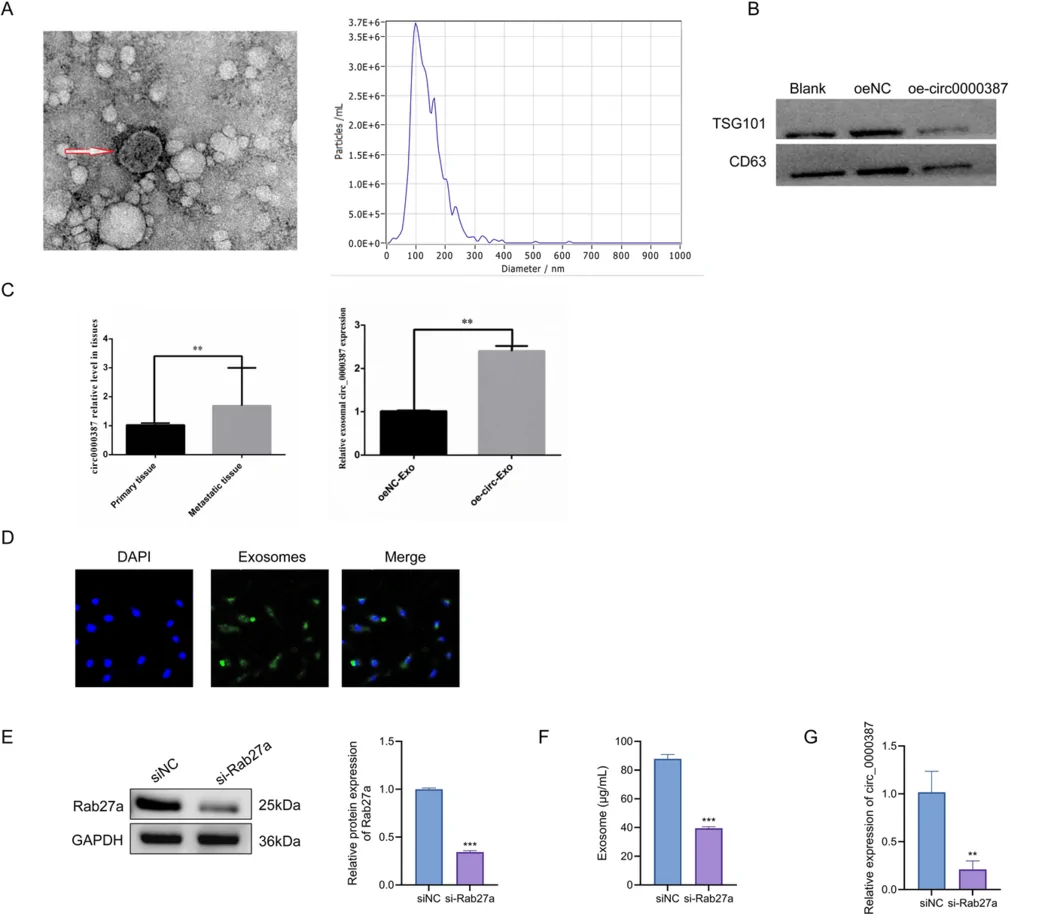
circ_0000387 is enriched in metastatic ovarian cancer tissues and SKOV3‐derived exosomes, and exosome‐associated fluorescence signals are observed in HUVECs. (A) Transmission electron microscopy (TEM) image (left) and nanoparticle tracking analysis (NTA) (right) of SKOV3‐derived exosomes. TEM and NTA were performed using three independent biological preparations (*n* = 3). (B) Western blot detection of the exosomal markers TSG101 and CD63. (C1–C2) qRT‐PCR analysis of circ_0000387 expression in paired primary versus metastatic ovarian cancer tissues and in SKOV3‐derived exosomes from oeNC versus oe‐circ cells, respectively. (D) Representative fluorescence images showing exosome‐associated signals in HUVECs after incubation with labelled SKOV3‐derived exosomes (DAPI, exosome signal, and merged images). (E) Western blot detection of Rab27a in siNC and si‐Rab27a cells. (F) ExoELISA assessment of relative CD63‐associated exosome abundance in conditioned medium. (G) qRT‐PCR analysis of exosomal circ_0000387 levels in the siNC and si‐Rab27a groups. **p < 0.01, ***p < 0.001 versus siNC.

qRT‐PCR analysis revealed significantly higher circ_0000387 levels in metastatic ovarian cancer tissues than in paired primary ovarian cancer tissues (*p* < 0.01, Figure 4C1). In SKOV3 cell‐derived exosomes, circ_0000387 abundance was higher in the oe‐circ group than in the oeNC group (*p* < 0.01, Figure 4C2).

Fluorescent imaging showed exosome‐associated fluorescence signals in HUVECs after incubation with labeled SKOV3‐derived exosomes (Figure 4D).

Rab27a knockdown was used to evaluate its role in exosome secretion. Western blotting confirmed a marked reduction in Rab27a protein levels in si‐Rab27a cells compared with siNC (*p* < 0.001, Figure 4E). Consistently, ExoELISA showed significantly lower relative CD63‐associated exosome abundance in conditioned medium from si‐Rab27a cells (*p* < 0.001, Figure 4F). qRT‐PCR further demonstrated reduced exosomal circ_0000387 levels in the si‐Rab27a group relative to siNC (*p* < 0.01, Figure 4G).

### 3.6 Exosomal circ_0000387 Increases Endothelial VWF Secretion and Reduces SKOV3 Viability, Invasion, and Migration

Given the established involvement of VWF in tumour–endothelial interactions, we next examined whether circ_0000387‐enriched exosomes altered endothelial VWF secretion in the SKOV3–HUVEC co‐culture system. ExoELISA analysis showed significantly higher relative CD63‐associated exosome abundance in conditioned medium from the oe‐circ group than from the oeNC group (*p* < 0.001). Both GW4869 and si‐Rab27a were associated with significantly lower relative CD63‐associated exosome abundance in conditioned medium than oe‐circ (*p* < 0.001) (Figure 5A). ELISA of HUVEC supernatants demonstrated that VWF levels were significantly upregulated in the oe‐circ group relative to oeNC (*p* < 0.001). GW4869 and si‐Rab27a each significantly decreased VWF secretion when compared with oe‐circ (*p* < 0.001) (Figure 5B). CCK‐8 assays showed that SKOV3 viability was significantly reduced in the oe‐circ group compared with oeNC (*p* < 0.001). Both GW4869 and si‐Rab27a significantly increased viability relative to oe‐circ (*p* < 0.001) (Figure 5C). Transwell invasion assays revealed that oe‐circ cells exhibited significantly reduced invasion compared with oeNC (*p* < 0.001). GW4869 and si‐Rab27a both significantly enhanced invasion relative to oe‐circ (*p* < 0.001) (Figure 5D). Wound‐healing assays showed decreased migration in the oe‐circ group relative to oeNC (*p* < 0.001). Migration was significantly increased in the oe‐circ+GW and oe‐circ+si‐Rab27a groups compared with oe‐circ (*p* < 0.001) (Figure 5E). Immunofluorescence analysis indicated that YAP nuclear localization was significantly reduced in the oe‐circ group compared with oeNC (*p* < 0.001). Both GW4869 and si‐Rab27a significantly increased nuclear YAP levels relative to oe‐circ (*p* < 0.001) (Figure 5F). Western blotting showed that p‐AKT was significantly decreased and p‐AMPK significantly increased in the oe‐circ group compared with oeNC (*p* < 0.001). GW4869 and si‐Rab27a both significantly increased p‐AKT and reduced p‐AMPK compared with oe‐circ (*p* < 0.001) (Figure 5G). Total AKT and AMPK levels remained unchanged.

**Figure 5:**
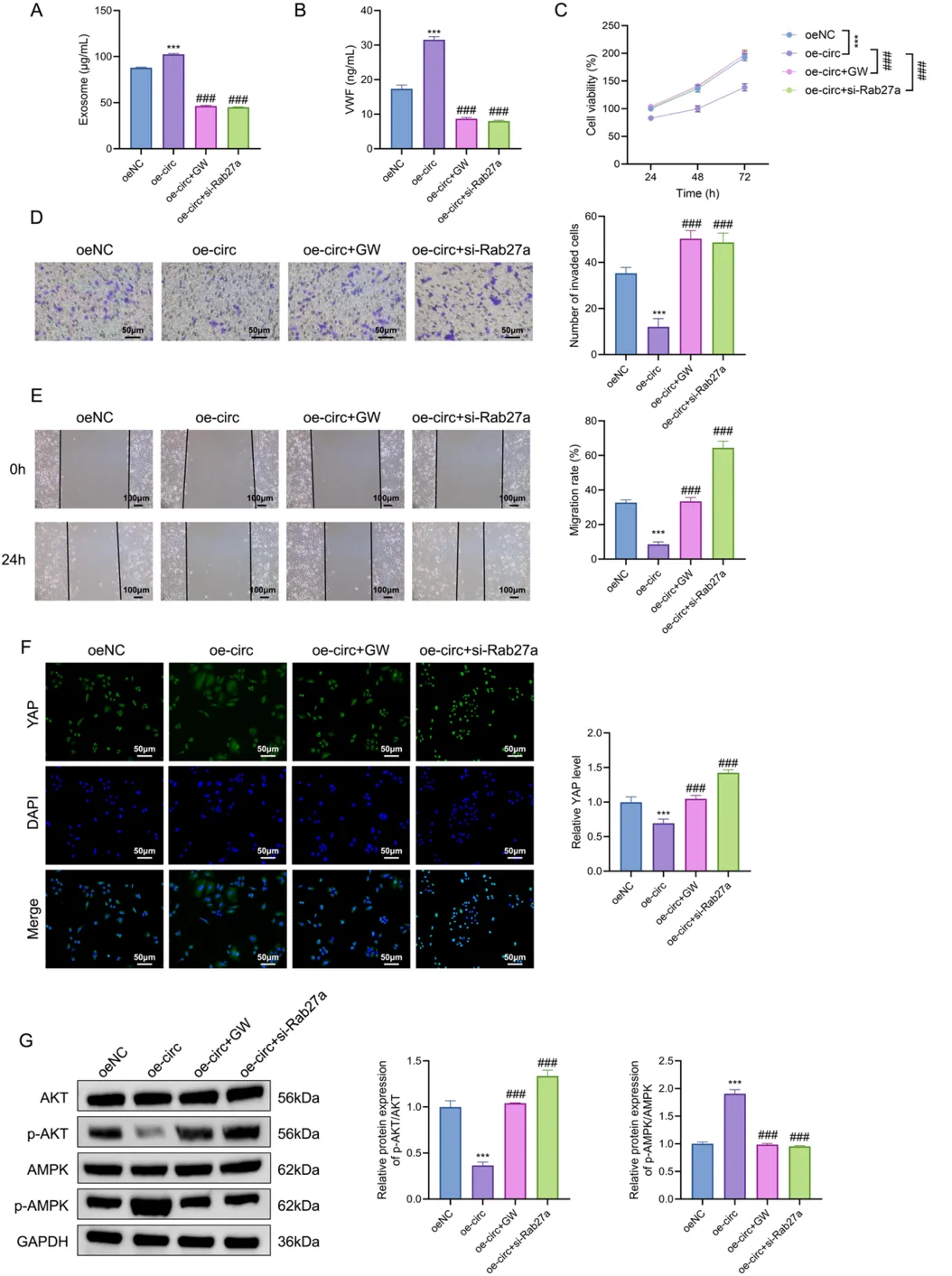
Exosome‐associated effects of circ_0000387 overexpression on VWF responses and SKOV3 cell phenotypes. (A) ExoELISA measurement of relative CD63‐associated exosome abundance in conditioned medium from oeNC, oe‐circ, oe‐circ+GW, and oe‐circ+si‐Rab27a groups. (B) ELISA assay of VWF levels in HUVEC supernatants. (C) CCK‐8 assay evaluating SKOV3 cell viability. (D) Transwell assay assessing SKOV3 invasion. Scale bar = 50 μm. (E) Wound‐healing assay evaluating SKOV3 migration. Scale bar = 100 μm. (F) Immunofluorescence staining of YAP subcellular localization. Scale bar = 50 μm. (G) Western blot detection of AKT, p‐AKT, AMPK, and p‐AMPK protein levels in SKOV3 cells. ***p < 0.001 versus oeNC; ^###^ *p* < 0.001 versus oe‐circ.

### 3.7 FVIII Modulates VWF‐Associated Responses in the Exosome Co‐Culture System

Given the functional association between VWF and FVIII, we next examined whether FVIII modified VWF‐associated endothelial and tumour‐cell responses in the exosome co‐culture system. ELISA results showed that VWF concentrations in HUVEC supernatants increased significantly upon co‐culture with SKOV3 cells under different treatments (Figure 6A, *P* < 0.01). Across all three conditions (blank, oeNC, oe‐circ), VWF levels increased following the addition of factor VIII (10–30 μg/mL), but the differences among groups at the same VIII concentration were small.

**Figure 6:**
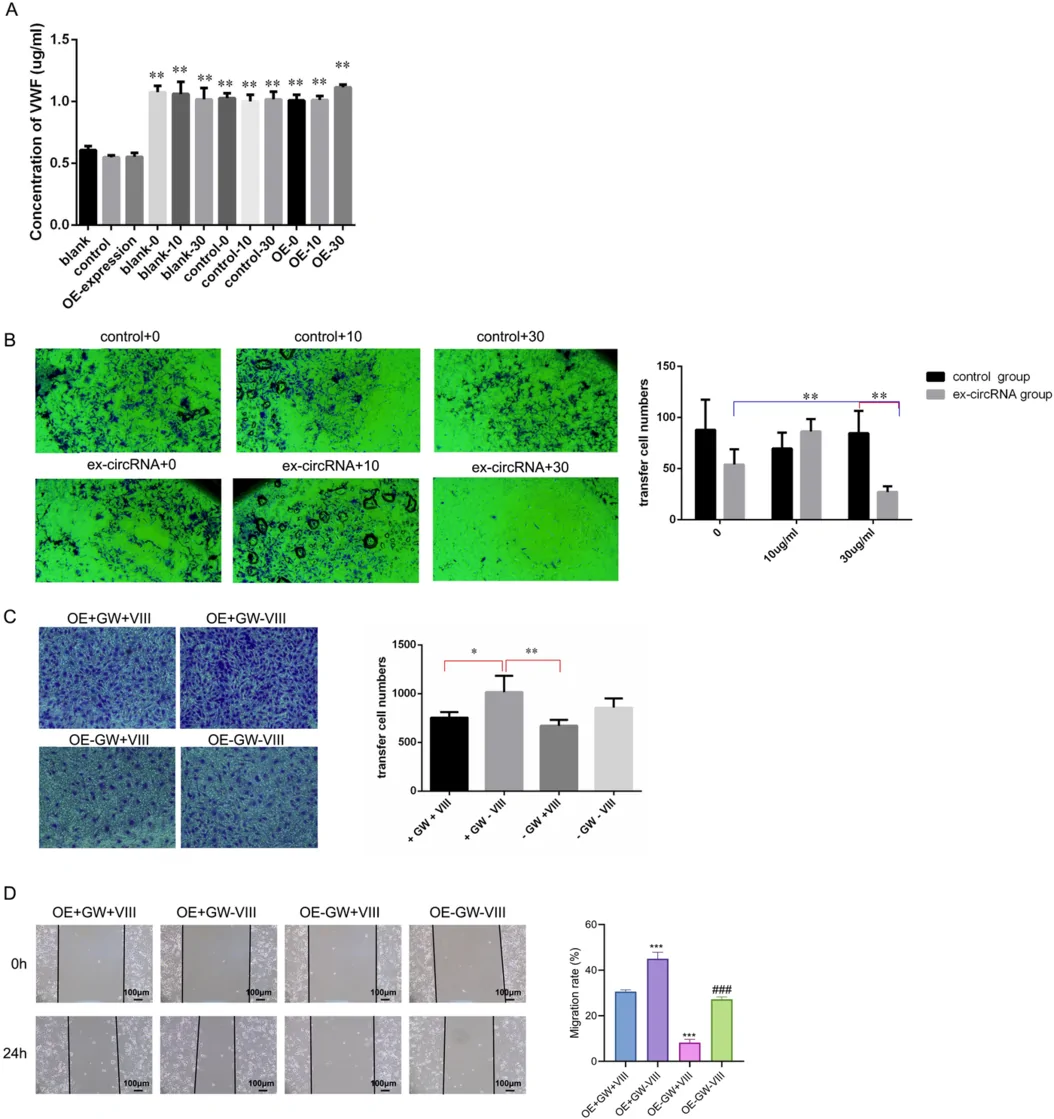
Effects of coagulation factor VIII on VWF secretion in HUVECs and on the invasion and migration of SKOV3 cells treated with exosomal circ_0000387. (A) ELISA measurement of VWF levels in HUVEC supernatants co‐cultured with blank, oeNC, or oe‐circ SKOV3 cells and treated with 0, 10, or 30 μg/mL factor VIII. (B) Transwell assay of SKOV3 invasion after co‐culture with HUVECs and treatment with the indicated concentrations of FVIII. (C) Transwell analysis of invasion in OE + GW + VIII, OE + GW–VIII, OE–GW + VIII, and OE–GW–VIII groups. (D) Wound‐healing assay assessing migration across the same four groups. Scale bar = 100 μm. *p < 0.05, **p < 0.01, ***p < 0.001 versus OE + GW + VIII; ^###^ *p* < 0.001 versus OE‐GW + VIII.

Transwell assays showed that SKOV3 invasion changed in response to FVIII treatment (Figure 6B). Without factor VIII, invasion was lower in the ex‐circ group than in the control group. At 10 μg/mL, invasion increased in both groups, particularly in the ex‐circ condition. At 30 μg/mL, however, invasion in the ex‐circ group declined sharply, falling below the 0 and 10 μg/mL levels.

In SKOV3‐derived exosome rescue experiments (Figure 6C), Transwell assays showed clear differences among the four conditions. In the absence of factor VIII, blocking exosome release (+GW–VIII) led to the highest invasion, significantly exceeding both +GW + VIII (*p* < 0.05) and –GW + VIII (*p* < 0.01). When exosome secretion was intact (–GW), the addition of factor VIII resulted in the lowest invasion, whereas –GW–VIII displayed an intermediate level. These findings suggest that intact exosome secretion in the presence of FVIII is associated with reduced invasion, whereas exosome blockade attenuates this effect.

Wound‐healing assays (Figure 6D) revealed clear differences among the four conditions. Migration was highest when exosome release was blocked in the absence of factor VIII (OE + GW–VIII; *p* < 0.001 vs OE + GW + VIII). When exosome secretion was intact, adding factor VIII (OE–GW + VIII) resulted in the lowest migration rate (*p* < 0.001 vs OE + GW + VIII), while OE–GW–VIII showed an intermediate level (*p* < 0.001 vs OE–GW + VIII). Similarly, intact exosome secretion in the presence of FVIII is associated with reduced migration, whereas exosome blockade attenuates this response.

### 3.8 VWF Knockdown Attenuates circ_0000387‐Associated Suppression of SKOV3 Migration and Invasion

ELISA results showed that VWF levels differed significantly across groups (Figure 7A). VWF secretion was markedly higher in oe‐circ+siNC cells compared with oeNC+siNC (*p* < 0.001). Knockdown of VWF significantly reduced VWF levels in both oeNC and oe‐circ conditions (*p* < 0.001), and VWF expression in oe‐circ+si‐VWF was significantly lower than in oe‐circ+siNC (*p* < 0.001).

**Figure 7:**
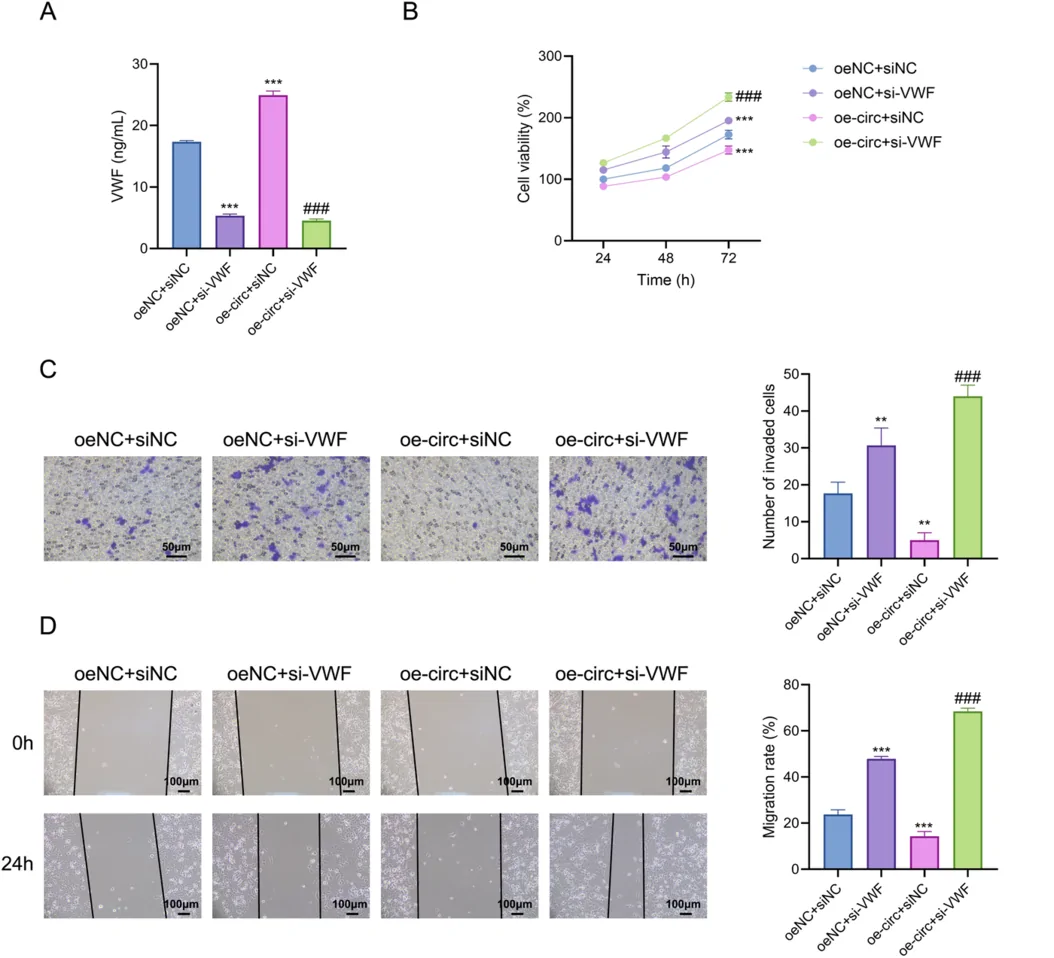
VWF contributes to the reduced migration and invasion associated with circ_0000387‐enriched exosome conditions. (A) ELISA analysis of VWF levels in HUVEC supernatants from oeNC+siNC, oeNC+si‐VWF, oe‐circ+siNC, and oe‐circ+si‐VWF groups. (B) CCK‐8 assay assessing cell viability across treatment groups. (C) Transwell invasion analysis in the four groups. Scale bar = 50 μm. (D) Wound‐healing assay evaluating migration ability. Scale bar = 50 μm. **p < 0.01, ***p < 0.001 versus oeNC+siNC; ^###^ *p* < 0.001 versus oe‐circ+siNC.

CCK‐8 assays demonstrated that cell viability was significantly reduced in the oe‐circ+siNC group compared with oeNC+siNC (*p* < 0.001) (Figure 7B). Knockdown of VWF significantly increased viability in the oe‐circ background (*p* < 0.001). oeNC+si‐VWF also showed increased viability relative to oeNC+siNC (*p* < 0.001).

Transwell assays showed that invasion was significantly decreased in the oe‐circ+siNC group compared with oeNC+siNC (*p* < 0.01) (Figure 7C). VWF knockdown significantly increased invasion in oe‐circ cells (*p* < 0.001). In the oeNC background, si‐VWF also significantly enhanced invasion compared with oeNC+siNC (*p* < 0.001).

Wound‐healing assays revealed significantly reduced migration in the oe‐circ+siNC group relative to oeNC+siNC (*p* < 0.001) (Figure 7D). Migration was significantly increased in oe‐circ+si‐VWF compared with oe‐circ+siNC (*p* < 0.001). In the oeNC condition, VWF knockdown also significantly increased migration compared with oeNC+siNC (*p* < 0.001).

### 3.9 Exosomal circ_0000387 Modulates Intraperitoneal Tumour Dissemination in Association With VWF and AKT/AMPK/YAP Signaling In Vivo

Representative abdominal images showed visible differences in peritoneal tumour burden across groups (Figure 8A). Quantification demonstrated that the number of peritoneal nodules was significantly reduced in OE‐circ mice compared with the Control group (*p* < 0.001). In contrast, both OE‐circ+si‐Rab27a and OE‐circ+anti‐VWF groups exhibited significantly higher nodule counts relative to OE‐circ (*p* < 0.0001, respectively) (Figure 8B).

**Figure 8:**
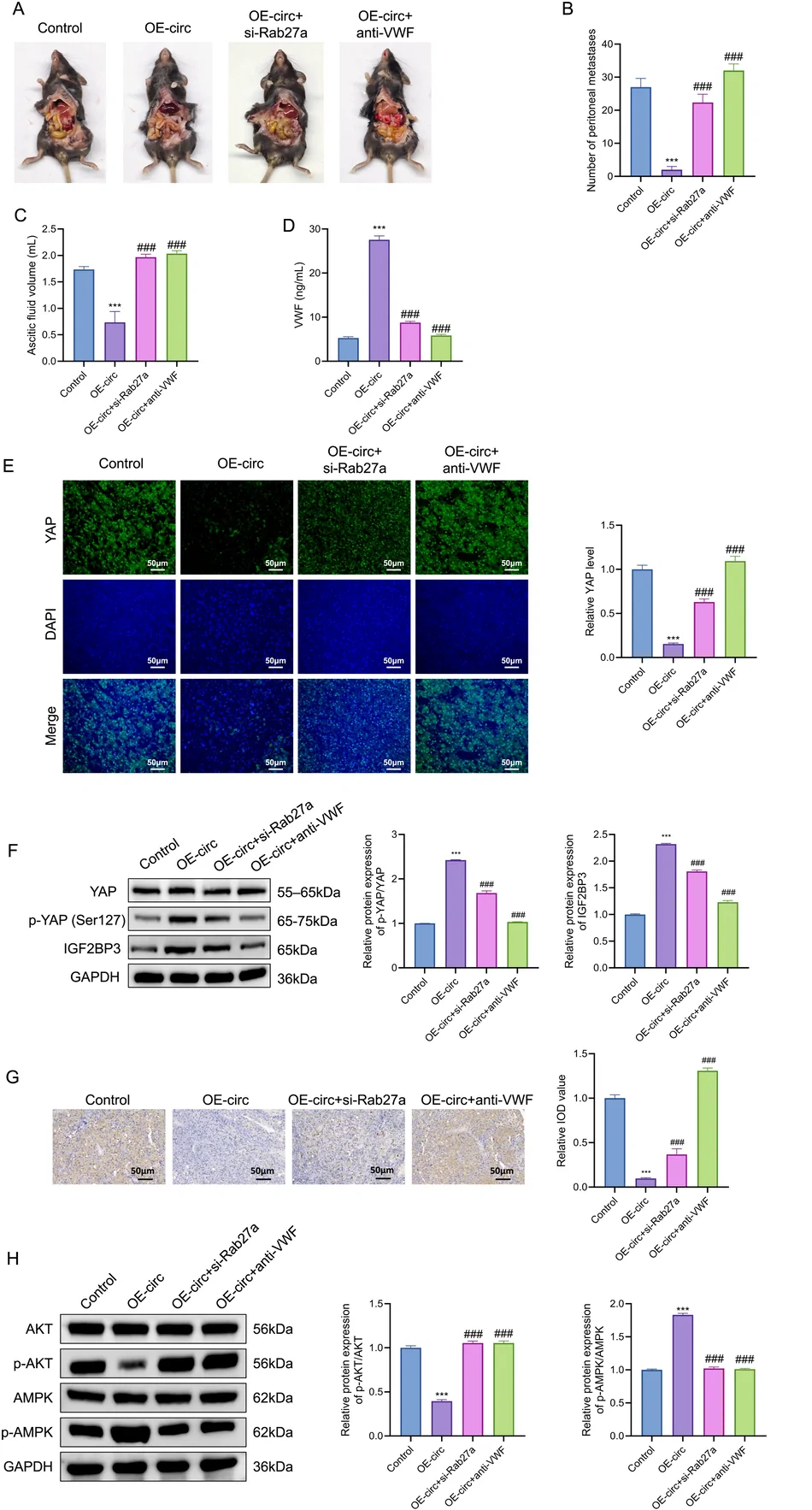
Exosome‐ and VWF‐associated effects of circ_0000387 overexpression on intraperitoneal dissemination and AKT/AMPK/YAP signalling in vivo. (A) Representative intraperitoneal tumour images. (B) Quantification of peritoneal metastatic nodules. (C) Measurement of ascites volume. (D) ELISA of plasma VWF levels. (E) Immunofluorescence staining of YAP localization in tumour sections. Scale bar = 50 μm. (F) Western blot analysis of YAP, p‑YAP (Ser127), and IGF2BP3 in tumour tissues. (G) Immunohistochemical staining of YAP in tumour sections. (H) Western blot detection of AKT, p‐AKT, AMPK, and p‐AMPK in tumour tissues. ***p < 0.001 versus Control; ^###^ *p* < 0.001 versus oe‐circ.

Ascites volume was significantly decreased in OE‐circ mice compared with Control (*p* < 0.001), whereas ascites in OE‐circ+si‐Rab27a and OE‐circ+anti‐VWF groups was significantly increased compared with OE‐circ (*p* < 0.001 for both) (Figure 8C).

Plasma VWF levels were significantly elevated in OE‐circ mice relative to Control (*p* < 0.001). VWF levels were significantly reduced in OE‐circ+si‐Rab27a and OE‐circ+anti‐VWF mice compared with OE‐circ (*p* < 0.001 for both) (Figure 8D).

Immunofluorescence staining revealed reduced YAP nuclear localization in OE‐circ tumours compared with Control (*p* < 0.001). YAP nuclear levels were significantly increased in both OE‐circ+si‐Rab27a and OE‐circ+anti‐VWF groups relative to OE‐circ (both *p* < 0.001) (Figure 8E).

To further assess YAP phosphorylation associated with its subcellular localization, western blot analysis was performed. The p‐YAP (Ser127)/YAP ratio was significantly increased in OE‐circ tumours compared with Control and was reduced by Rab27a silencing or VWF neutralisation (*p* < 0.001; Figure 8F). IGF2BP3 expression was also increased in OE‐circ tumours but decreased following either intervention (*p* < 0.001; Figure 8F). Consistent with the phosphorylation pattern, immunohistochemical staining showed reduced intratumoral YAP immunoreactivity in OE‐circ tumours, which was partially restored in the OE‐circ+si‐Rab27a and OE‐circ+anti‐VWF groups (*p* < 0.001; Figure 8G).

Western blot analysis showed that p‐AKT expression was significantly decreased in OE‐circ tumors compared with Control (*p* < 0.001), while p‐AMPK was significantly increased (*p* < 0.001). These changes were significantly reversed in OE‐circ+si‐Rab27a and OE‐circ+anti‐VWF groups, each showing higher p‐AKT and lower p‐AMPK levels than OE‐circ (*p* < 0.001 for both comparisons) (Figure 8H). Total AKT and AMPK remained unchanged across groups.

## 4 Discussion

This study systematically characterised the expression, cellular distribution, and functional roles of circ_0000387 in ovarian cancer. circ_0000387 was expressed at higher levels in metastatic ovarian cancer tissues than in paired primary ovarian cancer tissues and was also enriched in SKOV3 cell‐derived exosomes. Exosome‐associated fluorescence signals were detected in endothelial cells after incubation with labelled SKOV3‐derived exosomes. Functionally, intracellular circ_0000387 increased SKOV3 cell viability, invasion, and migration, accompanied by altered AKT and AMPK phosphorylation and enhanced YAP nuclear accumulation; these effects were associated with its interaction with IGF2BP3. In contrast, circ_0000387‐enriched exosomes increased endothelial VWF secretion and were associated with reduced SKOV3 invasion and migration in an FVIII‐modulated co‐culture context. Consistently, in vivo, circ_0000387 overexpression was associated with reduced intraperitoneal dissemination and ascites formation, and these effects were attenuated by Rab27a silencing or VWF neutralisation, together with corresponding changes in AKT/AMPK/YAP signalling. Collectively, these findings support a context‐dependent model in which circ_0000387 may exert pro‐tumour effects within ovarian cancer cells while being associated with anti‐metastatic activity through exosome‐dependent, VWF‐related tumour–endothelial communication.

Recent studies increasingly emphasize the importance of extracellular vesicles in bidirectional tumour–endothelial communication, particularly in shaping the metastatic microenvironment [19]. However, the contribution of exosomal circRNAs to endothelial regulation remains poorly understood. Our findings expand this area by demonstrating that circ_0000387 is enriched in exosomes and transferred to endothelial cells, thereby illustrating a compartment‐specific functional role for circRNAs in ovarian cancer. The opposing functions of circ_0000387 in tumour versus endothelial cells are consistent with emerging evidence that exosomal circRNAs exert context‐dependent effects. For instance, exosomal circTUBGCP4 and circPVT1 promote angiogenesis and metastasis in colorectal and laryngeal cancers, respectively [20, 21], whereas circFOXK2 suppresses angiogenesis in lung adenocarcinoma [22]. Thus, the dual functionality of circ_0000387 reflects the complexity of circRNA‐mediated intercellular communication, wherein the same molecule may produce opposite outcomes in different cell types due to distinct interacting partners and signaling environments. Further studies are needed to determine whether circ_0000387 directly regulates endothelial‐specific factors or indirectly induces VWF upregulation through broader exosomal signaling.

Previous work identified IGF2BP3 as an oncogenic RNA‐binding protein that enhances tumour survival and metastasis in several cancer types, including triple‐negative breast cancer and lung adenocarcinoma [23]. Yet, upstream non‐coding RNA regulators of IGF2BP3 remain insufficiently defined. In our study, CHIRP and RIP assays provided evidence of a physical interaction between circ_0000387 and IGF2BP3, suggesting that circ_0000387 may modulate IGF2BP3‐associated functions. Notably, although IGF2BP3 expression increased following circ_0000387 overexpression, the associated YAP‐related changes differed between the tumour‐cell‐intrinsic and in vivo settings, suggesting context‐dependent regulation. Combined with reports that IGF2BP3 preferentially binds m6A‐modified transcripts [24], these findings suggest that circ_0000387 may participate in IGF2BP3‐linked signaling processes, although the precise mode of regulation and its context dependence remain to be fully elucidated.

Furthermore, our study links circ_0000387 to coordinated modulation of the AKT, AMPK, and YAP pathways, which are central to tumour metabolism, growth, and mechanotransduction. Although prior studies have described crosstalk among these pathways during tumour invasion and metabolic stress [17], our findings revealed context‐dependent YAP regulation. circ_0000387 promoted YAP nuclear accumulation in SKOV3 cells, whereas in vivo circ_0000387 overexpression under exosome‐ and VWF‐dependent conditions was associated with reduced YAP nuclear localisation and increased p‐YAP (Ser127). These findings suggest a possible extension of this signaling framework to endothelial biology by showing that exosomal circ_0000387 increases VWF secretion in endothelial cells, a process associated with downstream changes in AKT, AMPK, and YAP signaling observed in co‐culture and in vivo. Together, these observations point to a multicellular interaction that links tumour‐intrinsic oncogenic signaling with extracellular microenvironment‐dependent anti‐metastatic cues.

The selection of VWF as an endothelial readout was guided by its established biological relevance to tumour–endothelial interactions rather than by the KEGG enrichment analysis. Beyond its canonical haemostatic function, endothelial‐derived VWF has been implicated in vascular permeability, tumour‐cell adhesion, transendothelial migration, and metastatic dissemination [13, 14]. Moreover, recent evidence indicates that small extracellular vesicle‐associated VWF can participate in reciprocal communication between tumour and endothelial cells [15], providing additional rationale for examining VWF in our exosome‐based co‐culture system. Following the observed increase in endothelial VWF secretion, FVIII was explored because VWF serves as its principal carrier and stabilising protein and because FVIII has been implicated in cancer‐associated pathophysiology [13, 16]. Our FVIII experiments should therefore be interpreted as exploratory evidence that FVIII can modify the VWF‐associated response under the present experimental conditions, rather than as demonstration of a canonical linear circ_0000387–VWF–FVIII signalling pathway.

Together, these findings suggest that circ_0000387 may exert compartment‐dependent effects in ovarian cancer, promoting malignant phenotypes within tumour cells while being associated with reduced metastatic activity through exosome‐dependent, VWF‐related endothelial responses. This context‐dependent behaviour highlights the complexity of circRNA‐mediated tumour–microenvironment communication and warrants further validation across additional ovarian cancer models.

While this study provides a comprehensive overview of the role of exosomal circ_0000387 in ovarian cancer progression, several aspects merit continued exploration. First, the functional and mechanistic findings were generated primarily using SKOV3 cells, and validation in additional ovarian cancer cell lines and disease‐relevant models is required to establish their generalisability. Second, the clinical cohort comprised only 20 patients from whom paired primary and metastatic tumour tissues were obtained; larger independent cohorts are therefore needed to validate the association between circ_0000387 expression and metastatic progression. Third, although an interaction between circ_0000387 and IGF2BP3 was demonstrated, the underlying regulatory mechanism remains unclear, including whether circ_0000387 affects IGF2BP3 through RNA stability, protein localisation, or other post‐transcriptional processes. In addition, exosome uptake by endothelial cells was assessed qualitatively, without defining the specific uptake routes or quantitatively measuring uptake efficiency. Importantly, whether the endothelial effects are directly attributable to transferred circ_0000387 rather than other exosomal cargo or broader exosome‐associated signalling remains unresolved; thus, the proposed circ_0000387‐associated exosome–VWF relationship should be regarded as a working model requiring further causal validation. Finally, validation in additional in vivo metastatic models would help establish the context dependence of these effects.

## 5 Conclusion

This study identifies circ_0000387 as a metastasis‐associated circRNA with context‐dependent functions in ovarian cancer. Intracellular circ_0000387 promoted malignant phenotypes in SKOV3 cells in association with IGF2BP3 and AKT/AMPK/YAP signalling, whereas circ_0000387‐enriched exosomes were associated with endothelial VWF responses and reduced tumour‐cell motility. In vivo findings further supported an exosome‐dependent and VWF‐associated effect on intraperitoneal dissemination. These findings highlight circ_0000387 as a potential regulator of both tumour‐intrinsic signalling and tumour–endothelial communication, although further mechanistic and multicentre clinical validation is required.

## Author Contributions

**Yulian Xie:** conceptualization, methodology, software, data curation, investigation, validation, formal analysis, supervision, funding acquisition, visualization, project administration, resources, writing – original draft, writing – review and editing. **Peijun Shen:** conceptualization, methodology, software, data curation, investigation, validation, formal analysis, supervision, funding acquisition, visualization, project administration, resources, writing – original draft, writing – review and editing. **Hailin Chen:** data curation, supervision, validation. **Qianqian Shen:** conceptualization, funding acquisition, validation. **Xiaoxia Tong:** formal analysis, supervision, data curation.

## Ethics Statement

This study was approved by the Ethics Committee of the Shanghai Zhongshan Hospital Qingpu Branch (Approval No: Qingyi2022‐39). All animal experiments were reviewed and approved by the Ethics Committee of Luodian Hospital, Baoshan District, Shanghai (Approval No. LDYY‐KY‐2025‐01), and were conducted in accordance with institutional guidelines for the care and use of laboratory animals.

## Conflicts of Interest

The authors declare no conflicts of interest.

## Supporting information


Supporting File

## Data Availability

The data used to support the findings of this study are available from the corresponding author upon request.
